# Lung Metastasis Probability in Ewing Sarcoma: A Nomogram Based on the SEER Database

**DOI:** 10.3390/curroncol28010009

**Published:** 2020-12-05

**Authors:** Jie Wang, Yonggang Fan, Lei Xia

**Affiliations:** Department of Orthopaedic Surgery, The First Affiliated Hospital of Zhengzhou University, Zhengzhou 450052, China; 13027709327@163.com (J.W.); fanyonggang1996@163.com (Y.F.)

**Keywords:** Ewing sarcoma, SEER database, lung metastasis, nomogram

## Abstract

Background. Up to now, an accurate nomogram to predict the lung metastasis probability in Ewing sarcoma (ES) at initial diagnosis is lacking. Our objective was to construct and validate a nomogram for the prediction of lung metastasis in ES patients. Methods. A total of 1157 patients with ES from the Surveillance, Epidemiology, and End Results (SEER) database were retrospectively collected. The predictors of lung metastasis were identified via the least absolute shrinkage and selection operator (LASSO) and multivariate logistic analysis. The discrimination and calibration of the nomogram were validated by receiver operating characteristic (ROC) curve and calibration curve. Decision curve analysis (DCA) was used to evaluate the clinical usefulness and net benefits of the prediction model. Results. Factors including age, tumor size, primary site, tumor extension, and other site metastasis were identified as the ultimate predictors for the nomogram. The calibration curves for the training and validation cohorts both revealed good agreement, and the Hosmer–Lemeshow test identified that the model was well fitted (*p* > 0.05). In addition, the area under the ROC curve (AUC) values in the training and validation cohorts were 0.732 (95% confidence interval, CI: 0.607–0.808) and 0.741 (95% CI: 0.602–0.856), respectively, indicating good predictive discrimination. The DCA showed that when the predictive metastasis probability was between 1% and 90%, the nomogram could provide clinical usefulness and net benefit. Conclusion. The nomogram constructed and validated by us could provide a convenient and effective tool for clinicians that can improve prediction of the probability of lung metastasis in patients with ES at initial diagnosis.

## 1. Introduction

Ewing sarcoma (ES) is the second most common malignant primary osseous neoplasm, accounting for 8% of all cases in children and adolescents [1,2]. With the development of multidisciplinary therapy, the 5-year overall survival (OS) of ES has gradually improved from 10% to 75% [3]. Despite the proven effectiveness of the treatment of localized disease, the 5-year OS of ES patients with metastasis is below 30%, suggesting that these patients still fare poorly [4]. It is worth noting that most patients already have micrometastases at initial diagnosis [5]; however, only 20–28% of patients present with metastasis at initial diagnosis, and the most common site is the lung (50%) [4,6]. Although patients with lung metastasis alone have better survival than those with metastases at other sites, their mortality at 5 years is still approximately 60–70% [7,8,9,10]. The survival outcomes of patients with multiple metastases within the lung are even worse [11]. The early and accurate diagnosis of metastasis is of great significance for the targeted treatment of ES [12]. Nevertheless, because of the characteristics of micrometastases and the insufficient ability of current radiological techniques (multidetector row CT) to detect small lung nodules [13,14], improving the accuracy in detecting lung metastasis at initial diagnosis is necessary.

Some studies have investigated potential risk factors for metastasis to facilitate early diagnosis [11,15]. However, these studies analyzed only as single factor to evaluate metastasis in patients with ES. A predictive tool such as a nomogram, which can integrate multiple significant risk features to comprehensively predict lung metastasis probability, is urgently needed. Nomograms have been confirmed to provide superior individual disease risk estimation and enable accurate treatment decisions [16].

We analyzed the Surveillance, Epidemiology, and End Results (SEER) database, which collects data from seventeen geographically variable cancer registries and represents approximately 26% of the U.S. population [17], to identify independent risk factors for lung metastasis in ES at initial diagnosis; in addition, we constructed and validated a nomogram to predict lung metastasis probability.

## 2. Materials and Methods

### 2.1. Patient Cohort

The inclusion criteria were as follows: (1) diagnosed as ES of the bones with ICD-O-3/WHO 2008 morphology codes 9260 after 2010 from the SEER database; (2) microscopically confirmed, positive histology confirmed or positive exfoliative cytology confirmed.

The exclusion criteria were as follows: (1) unknown metastasis status; (2) unknown race; (3) unknown tumor size.

The clinicopathological features of the patients were categorized as follows: (1) age (<20 years old, 20 to 50 years old and >50 years old), sex (male or female), race (white, black, or other (Native American/Alaskan Native or Asian/Pacific Islander)); (2) tumor size (<5 cm, 5 to 10 cm, or >10 cm), tumor extension (inside the periosteum or beyond the periosteum), primary site (extremity (long or short bones of the upper or lower extremities), axial (skull, pelvis, spine, or ribs) or other locations), and metastasis (lung metastasis or other site metastasis).

No personal identifying information was used in the study. Hence, we did not require Institutional Review Board approval or patient informed consent. Informed consent was not required because of the retrospective nature of the study.

### 2.2. Statistical Analysis

We randomly divided all patients (*n* = 1157) into a training cohort (*n* = 812) and a validation cohort (*n* = 345). The baseline clinicopathological features were compared via the chi-square test between the two groups. To select the initial factors and prevent overfitting of the multifactor models, least absolute shrinkage, and selection operator (LASSO) regression was performed [18]. Furthermore, we used multivariate logistic regression to identify the ultimate predictive factors for the nomogram.

Using the training and validation cohorts, we validated the nomogram internally and externally. The predictive discrimination of the nomogram was assessed via a receiver operating characteristic (ROC) curve and the area under the curve (AUC), and the concordance of the nomogram was validated with a calibration plot and the Hosmer–Lemeshow test. Moreover, we utilized decision curve analysis (DCA) to assess the clinical usefulness and net benefits of the nomogram [19,20].

The chi-square test was performed via SPSS statistics software version 22.0 (IBM Corporation, Armonk, NY, USA), and the remaining statistical analyses were performed and the graphics generated by R software (3.6.3) and R studio software (1.2.5033). A two-sided *p* value < 0.05 was considered to have statistical significance.

## 3. Results

According to the inclusion and exclusion criteria, a total of 1157 ES patients, which were assigned to the training cohort (*n* = 812, for the construction and internal validation of the nomogram) or the validation cohort (*n* = 345, for the external validation of the nomogram), were identified. Most of the patients were below 20 years old, and the total proportion of patients with lung metastasis at initial diagnosis was 10.2% (Table 1). The chi-square test showed no significant differences between the two cohorts in lung metastasis, age, sex, race, tumor size, tumor extension, primary site, or other site metastasis (Table 1, *p* > 0.05).

**Figure 1:**
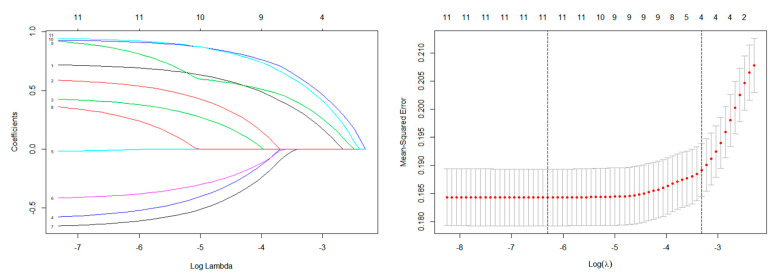
The results of the least absolute shrinkage and selection operator (LASSO) regression.

To avoid overfitting, the LASSO regression selected six features with nonzero coefficients when lung metastasis was the endpoint, including age, race, tumor size, tumor extension, other site metastasis and primary site in the training cohort (Figure 1). The multivariate logistic regression analysis demonstrated that age (>50 years old, OR = 2.059, 95% CI = 1.459–4.886, *p* = 0.003), tumor size (5–10 cm, OR = 2.620, 95% CI = 1.494–4.823, *p* = 0.003; >10 cm, OR = 1.478, 95% CI = 0.814–2.800, *p* = 0.000), primary site (Axial, OR = 1.535, 95% CI = 1.064–2.218, *p* = 0.022), tumor extension (beyond periosteum, OR = 0.398, 95% CI = 0.269–0.581, *p* = 0.000) and other site metastasis (yes, OR = 2.610, 95% CI = 1.677–4.072, *p* = 0.000) were independent risk factors for lung metastasis in patients with ES (Table 2).

**Figure 2:**
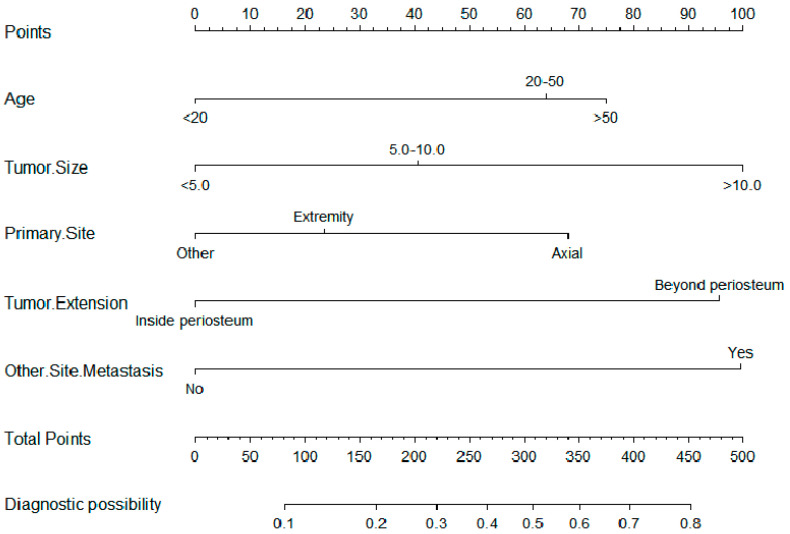
The nomogram for predicting the probability of lung metastasis.

**Figure 3:**
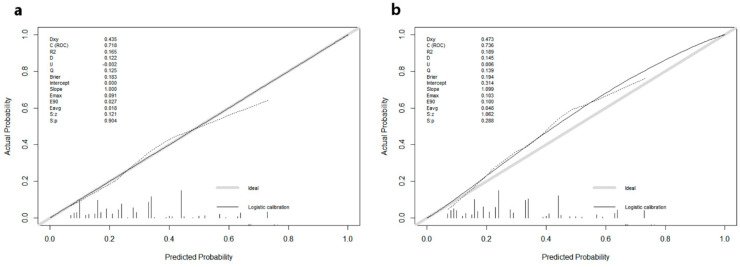
Calibration curves for the training (**a**) and validation (**b**) cohorts.

**Figure 4:**
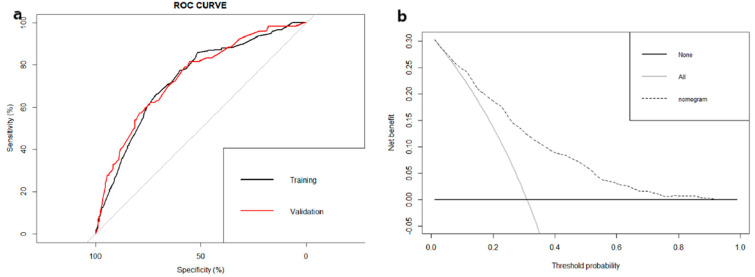
The area under the ROC curve (AUC) values in the training and validation cohorts were 0.732 (95% CI: 0.607–0.808) and 0.741 (95% CI: 0.602–0.856), respectively (**a**), indicating good predictive discrimination. The decision curve analysis (DCA) showed that when the predictive metastasis probability was between 1% and 90%, the nomogram could provide clinical usefulness and net benefit (**b**).

The nomogram was constructed and is presented in Figure 2. The calibration curves for the training (Figure 3a) and (Figure 3b) validation cohorts both revealed good agreement, and the Hosmer–Lemeshow test identified that the model was well fitted (*p* > 0.05). In addition, the area under the ROC curve (AUC) values in the training and validation cohorts were 0.732 (95% CI: 0.607–0.808) and 0.741 (95% CI: 0.602–0.856), respectively (Figure 4a), indicating good predictive discrimination. The DCA showed that when the predictive metastasis probability was between 1% and 90%, the nomogram could provide clinical usefulness and net benefit (Figure 4b).

## 4. Discussion

Lung metastasis in patients with ES can be affected by multiple risk factors [11,15,21,22,23]. Pathways related to platelet-derived growth factor (PDGF) signaling, Wnt signaling, apoptosis signaling, TP53, Notch signaling, and angiogenesis have been found to be of importance for the occurrence and development of metastasis in ES. Some genes have also been identified to contribute to the lung metastasis of ES. Na et al. found that CXC-chemokine receptor 6 (CXCR6) and CXC-chemokine ligand 16 (CXCL16) expression in tumor cells significantly correlated with a central location and the occurrence of lung metastasis [23]. Von et al. reported that chondromodulin 1 (CHM1) expression was increased in patients with ES lung metastases [22]. However, the clinical risk factors that affect lung metastasis in patients with ES have not been fully described. Previous clinical studies have mainly investigated all metastasis rather than lung metastasis at initial diagnosis [11,15]. In addition, previous studies did not integrate these factors, instead focusing on a single predictive index, which may have a limited effect on predicting an individual instance of lung metastasis. In recent years, nomograms have been recognized as efficient tools that can integrate all independent risk factors for diagnosis or survival outcome [24,25]. However, previous nomograms associated with ES only estimated individual patient survival outcomes, and a nomogram to predict lung metastasis in patients with ES has not yet been reported. Thus, we generated a novel nomogram to fulfill this aim. To our knowledge, this is the first study to describe a nomogram to predict lung metastasis in patients with ES.

In this study, LASSO regression and multivariate logistic regression analyses were performed to screen for risk factors and to identify independent risk factors. Variables, including age at diagnosis, tumor size, tumor extension, primary site, and other site metastasis, were independent risk factors for lung metastasis in patients with ES. As an independent risk factor, the influence of age on metastasis has been investigated in previous research findings. Ye et al. reported that ES patients between 18 and 59 years old had a high likelihood of metastatic disease at initial diagnosis [11]. Karski et al. and Ramkumar et al. found that advanced age may increase the metastasis probability of ES [26,27]. Our analyses also demonstrated that age beyond 50 years old was an independent risk factor for lung metastasis (OR = 2.059, 95% CI = 1.459–4.886, *p* = 0.003).

In addition, we also found that large tumor size was an independent predictor for the presentation of lung metastasis in ES patients at initial diagnosis. Large tumor size has been consistently reported as a contributor to the poor prognosis of ES patients [7,15,28,29], and it also has a major influence on metastasis in ES. Hense et al. identified that increased tumor size was positively associated with metastasis in patients with ES [30]. Ramkumar et al. showed that a tumor size greater than 118 mm caused the metastasis risk in ES patients to triple [27]. Analogously, tumors larger than 80 mm were confirmed to be more likely to have metastasis by Ye et al. [11]. Considering that increased tumor size can increase the difficulty in entirely removing the tumor and acquiring proper margins, this relationship between large tumors and metastasis seems logical. In addition, we found that tumors with a primary site in axial bones were more likely to have metastatic diseases at initial diagnosis than tumors with primary sites in other locations, which was also supported by previous results [11,15,27]. Given their nature, axial tumors are more likely to extend into the visceral cavities, thus resulting in noticeable symptoms later than tumors at other locations [31,32]. In such cases, when patients notice relevant symptoms and go to the hospital, the tumors usually are already large, and distant metastasis may have already occurred.

In the present study, the other identified predictor of lung metastasis was tumor extension. Tumor extension beyond the periosteum generally means higher malignancy and higher odds of distant metastasis. In addition, in the lung metastasis subgroup of this study, approximately 37.3% (44/118) of patients had other site metastasis at initial diagnosis. Once multiple metastases occur, metastases in the lung become very likely [6,11]. Thus, regarding metastasis at other sites as a predictive factor for lung metastasis is rational and necessary.

Undoubtedly, compared with general treatment, personalized treatment is more rational and specific [33]. As a concise but visualizable predictive model, a nomogram can be tailored according to the individual profile of the patient [34]. Such predictive tools can help clinicians optimize early diagnosis and develop personalized treatment strategies. For example, consider a 60-year-old ES patient with a tumor greater than 10 cm and tumor extension beyond the periosteum with a primary tumor site in the spine. For this patient, we could use the nomogram to connect each risk factor and obtain the patient’s total points (Figure 2). By adding up the points of each risk factor, we would obtain his ultimate score of 345 and thus conclude his lung metastatic probability is approximately 60%. According to the DCA, our nomogram would provide clinical usefulness and net benefit for our patient, as his metastasis probability is within the range of 1% to 90% (Figure 4b). Based on his result from the nomogram, we may advise that the patient be monitored for lung metastasis and consider performing detailed examinations, such as high-resolution CT or PET/CT, if necessary [35].

It is also important to consider the potential limitations of the present study. First, the retrospective nature of this study may have resulted in potential bias. Second, we validated the nomogram internally and externally with data from the same center, and, if possible, the nomogram should be validated with data from different centers to be more reliable. Finally, the SEER database did not include variables such as tumor markers and the expression of genes. Future studies could try to add these factors and develop a more comprehensive predictive model for lung metastasis of ES.

## 5. Conclusions

A nomogram to predict lung metastasis in patients with ES was constructed and validated based on independent factors, including age, tumor size, tumor extension, primary site, and other site metastasis. We believe this nomogram is a convenient and effective tool for clinicians that can improve prediction of the probability of lung metastasis in patients with ES at initial diagnosis.

## Figures and Tables

**Table 1:** Distribution of demographic and clinical information.

Variables	Total Population (N = 1157; 100.0%)		Training Cohort (N = 812; 70.1%)		Validation Cohort (N = 345; 29.9%)		*p*-Value
	N	%	N	%	N	%	
Lung Metastasis							0.616
No	1039	89.8	716	88.2	308	89.3	
Yes	118	10.2	96	11.8	37	10.7	
Age (years)							0.376
20	751	64.9	537	66.1	214	62.0	
20–50	336	29.0	229	28.2	107	31.0	
50	70	6.1	46	5.7	24	7.0	
Race							0.619
White	1029	88.9	726	89.4	303	87.8	
Black	44	3.8	31	3.8	13	3.8	
Other	84	7.3	55	6.8	29	8.4	
Sex							0.719
Male	722	62.4	504	62.1	218	63.2	
Female	435	37.6	308	37.9	127	36.8	
Primary Site							0.893
Axial	406	35.1	287	35.3	119	34.5	
Extremity	506	43.7	356	43.8	150	43.5	
Other	245	21.2	169	20.8	76	22.0	
Tumor Size(cm)							0.088
<5	176	15.2	113	13.9	63	18.3	
5–10	410	35.4	284	35.0	126	36.5	
>10	571	49.4	415	51.1	156	45.2	
Tumor Extension							0.160
Inside periosteum	397	34.3	289	35.6	108	31.3	
Beyond periosteum	760	65.7	523	64.4	237	68.7	
Other Sites Metastases							0.233
No	991	85.7	702	86.5	289	83.8	
Yes	166	14.3	110	13.5	56	16.2	

Chi-square test: these values are statistically significant at a *p* value of < 0.05.

**Table 2:** Multivariate logistic regression for analyzing the metastasis associated factors in the training cohort.

Variables	Training Cohort (N = 812)	
	OR (95% CI)	*p*-Value
Age		
20	1 (reference)	
20–50	1.852 (0.944–5.320)	0.068
50	2.059 (1.459–4.886)	0.003 *
Race		
White	1 (reference)	
Black	0.352 (0.120–1.013)	0.075
Other	0.640 (0.288–1.463)	0.053
Tumor Size(cm)		
5	1 (reference)	
5–10	2.620 (1.494–4.823)	0.001 *
10	1.478 (0.814–2.800)	0.000 *
Primary Site		
Other	1 (reference)	
Extremity	0.798 (0.496–1.267)	0.344
Axial	1.535 (1.064–2.218)	0.022 *
Tumor Extension		
Inside periosteum	1 (reference)	
Beyond periosteum	0.398 (0.269–0.581)	0.000 *
Other Sites Metastases		
No	1 (reference)	
Yes	2.610 (1.677–4.072)	0.000 *

Multivariate logistic regression: these values are statistically significant (*) at a *p* value of < 0.05.

## Abbreviations

ES	Ewing sarcoma
OS	overall survival
SEER	the Surveillance, Epidemiology, and End Results
LASSO	least absolute shrinkage and selection operator
ROC	receiver operating characteristic
AUC	area under the curve
DCA	decision curve analysis
CXCR6	CXC-chemokine receptor 6
CXCL16	CXC-chemokine ligand 16
CHM1	chondromodulin 1
